# The past, present and future of anticholinergic drugs

**DOI:** 10.1177/20451253231176375

**Published:** 2023-09-08

**Authors:** David Healy

**Affiliations:** Data Based Medicine Americas Ltd., 95 Sandringham Drive, Toronto, ON, M3H 1E1 Canada

**Keywords:** anticholinergic drugs, cognitive burden, confusion, deliriants, deprescribing, peripheral neuropathy

## Abstract

In current medical practice, it is difficult to find any reports claiming that drugs that are primarily anticholinergic or those that have significant anticholinergic effects have any therapeutic benefits. These drugs fell into disrepute within the mental health field from the mid-1960s onwards, and their supposed problems extended to elsewhere in medicine after that. There is considerable evidence that this disrepute stemmed more from marketing copy rather than from hard clinical trial data. Many apparent reviews appear to repeat prior claims rather than present substantial or new evidence. This article offers a perspective rather than a systematic review as there is little evidence other than claims to review. The aim is to challenge the conventional narrative that anticholinergic effects are uniquely hazardous by pointing to the uncertain basis for claims about the harms of anticholinergic drugs, antimuscarinic drugs in particular, ending with pointers to recent research that, if realized, might underpin important possible future benefits.

## Introduction

Acetylcholine was famously the first discovered neurotransmitter.^1^ It was followed soon after by histamine. From the 1920s through to the 1950s, drug development was dominated by these two transmitters, in part because of demonstrable effects on these systems of anticholinergic and antihistamine drugs already in use. The discovery of the sympathetic hormones, adrenaline and noradrenaline, led to medicines active on catecholamine receptors. Enteramine, later called serotonin, was discovered in the 1930s, but it offered few therapeutic options before the 1970s.

The late 19th-Century discovery that what are now known to be anticholinergic drugs were effective in Parkinson’s disease supported the development of other anticholinergic drugs. This led to discoveries that the cholinergic system had nicotinic and muscarinic arms, and that in the body, the sympathetic system was balanced, in an autonomic nervous system, by a parasympathetic system in which acetylcholine was the main neurotransmitter. Acetylcholine is also the main neurotransmitter of the Vagus nerve, a predominantly sensory component of the autonomic system, which regulates heart, gut and most visceral systems.

It is now clear that many anticholinergic and antihistamine drugs available in the 1950s also had actions on the serotonin and noradrenaline systems, with the antihistamines giving rise to most modern antipsychotic and antidepressant drugs including the selective serotonin reuptake inhibitors (SSRIs).^2^

A number of antinicotinic drugs – doxacurium, hexamethonium and mecamylamine – are used regularly as ganglion-blocking agents to relax or paralyse muscles for anaesthesia.^3^ These medicines are used acutely, are reversible and their use has not come under a cloud.

This is not the case with the antimuscarinic effects of both primarily anticholinergic and other drugs. Some of these agents, such as pirenzepine, remained a mainstay of treatments for gastric ulcers through to the 1980s, with others in continuing use for chronic obstructive pulmonary disease, Parkinson’s disease, overactive bladder, spasmodic gut disorders, hyperhidrosis and hypersalivation.

More generally, however, in the last decade, efforts to reduce medication burdens (deprescribing) have focused on an antimuscarinic burden as a target for action.^4–9^ Reducing medication burdens is one of the most pressing needs of the modern era. There are few safe paths through the deprescribing maze. Antimuscarinic burden appears to many as a safe option, but this approach risks running into problems if the anticholinergic effects of medicines are not the problem we have been led to believe.

This article reviews the origins of the anticholinergic problem, challenges the reputation these drugs currently have and points towards future therapies. It is necessarily more an opinion piece than a systematic review, as while there are many articles on this issue, there is little good data underpinning many of claims about the problems anticholinergic effects pose.

## The origins of an anticholinergic problem

The discovery of noradrenaline in the central nervous system in 1954^10^ opened up the possibility of linking catecholamines to behaviour. Interest grew rapidly when reserpine, an antihypertensive drug and tranquilizer, was demonstrated in 1955 to deplete noradrenaline, serotonin and later dopamine.^11,12^

These findings and the demonstration in 1961 of a noradrenaline reuptake mechanism on which tricyclic antidepressants (TCAs) worked, allied to which was evidence that monoamine oxidase inhibitors prevented the breakdown of catecholamines, laid the basis in 1965 for the catecholamine hypothesis of depression, according to which mood disorders stemmed from a depletion of noradrenaline and treatment involved a restoration of appropriate noradrenaline levels.^13^ The catecholamine hypothesis remained the dominant hypothesis for two decades, and only lost ground in both the academic and public imagination with the emergence of the SSRIs.

This catecholamine hypothesis followed on the heels of a demonstration that dopamine was depleted in Parkinson’s disease. Combined these findings suggested that neurological and psychiatric disorders might be inborn errors of metabolism or at least single neurotransmitter disorders.^14^ This view was superseded around 1970 as the focus shifted to adrenergic receptors.^2^

The main group of antidepressants in the 1960s were the TCAs. It is now known that these inhibit noradrenaline and serotonin reuptake, in addition to being anticholinergic, and having various other actions on histamine, catecholamine and serotonin systems. The catecholamine hypothesis called for pure catecholamine reuptake inhibitors, freed from any ancillary actions such as their anticholinergic or serotonergic effects. Pure drugs, it was implied, would be more effective and free of side effects.

Against this backdrop, the anticholinergic actions of the TCAs were painted as causing urinary retention, constipation, blurred vision, a dry mouth, tachycardia, falls, confusion and other problems. There was little appreciation at the time that an anticholinergic effect might be a therapeutic principle in its own right, offering benefits in mood disorders and other conditions.^2^

By the 1990s, the idea that anticholinergic drugs are good for nothing becomes clear in proposals that SSRIs only appear to work in clinical trials because they cause side effects, which alert patients to the fact they are on an active drug producing a placebo effect. The way suggested to eliminate this methodological issue was to compare SSRIs with active placebos. The anticholinergic group of drugs were proffered as the ideal active placebo – a set of drugs that only cause side effects without any known benefit.^15^

Another factor was that the catecholamine hypothesis of depression and dopamine hypothesis of schizophrenia put a premium on viewing the major psychiatric syndromes as single neurotransmitter disorders. Mood disorders were linked to catecholamines, psychosis to dopamine and anxiety to serotonin. This left dementia to acetylcholine. There was a plausible basis to link acetylcholine and dementia given evidence that atropine and scopolamine (hyoscine) can cause memory problems.^16^

The proposed link between cholinergic systems and cognitive function is also reflected in the Beers criteria, from 1991, which defined anticholinergic burden as the single biggest iatrogenic problem in the treatment of the elderly.^17^ Most subsequent efforts to promote deprescribing have adopted the Beers criteria and aimed at reducing a patient’s anticholinergic burden.

In addition, companies bringing new drugs on the market, such as the SSRIs, have pointed to their freedom of anticholinergic effects as a benefit. This attribution is not limited to antidepressants. For example, a recent review of anticholinergic drugs, such oxybutynin, used for overactive bladder disorders states that in some older patients, these can lead in short order to dementia, and therefore new drugs, such as mirabegron, should be used.^18^ Other reviews have taken a similar position without providing clear evidence that the anticholinergic effects are more problematic than dopaminergic, serotonergic or gaba-ergic effects.^19^

In the 1980s, tardive dyskinesia (TD) was one of the most serious problems that psychotropic drugs caused. This was linked to the antipsychotic drugs, which have a primary action on dopamine systems. Rather than attribute TD to actions on the dopamine system, there was a move to blame anticholinergic drugs and limit their use.^20^

As a result, there are comparatively few primarily antimuscarinic drugs in use today. The anticholinergic burden people supposedly suffer from now stems from a combination of drugs that have some antimuscarinic effects, such as antidepressants and antipsychotics, rather than exposure to medicines optimized for anticholinergic effects. As many drugs have some anticholinergic effects, an anticholinergic burden may equate to a medication burden, with other actions of these drugs in fact causing the problems attributed to anticholinergic actions.

## Problems with the anticholinergic problem

There are a number of problems with blaming the anticholinergic actions of current medications for all the problems it is claimed they cause.

Even before the catecholamine hypothesis, imipramine, the first tricyclic antidepressant, was being used to manage nocturnal enuresis in children. With the formulation of the catecholamine hypothesis, this benefit was attributed to imipramine’s antimuscarinic action.

Had an antimuscarinic effect been the primary mechanism of action underpinning a benefit for enuresis, it would have made more sense to use a primarily anticholinergic drug, but these are not as effective as imipramine. It later became clear that catecholamine reuptake inhibitors with minimal or no actions on the cholinergic system, such as reboxetine, and duloxetine, more potently trigger urinary retention than primarily antimuscarinic drugs. Drugs active on the catecholamine system, without any cholinergic effects, are now among the most commonly used medicines in the management of urinary flow issues.

Duloxetine, a noradrenaline and serotonin reuptake inhibitor is licensed for bladder stabilization in Europe. The conditions it treats are more often called interstitial cystitis than neurogenic bladder, for which anticholinergic agents were supposedly indicated. Interstitial cystitis and related painful conditions can be both caused by serotonin reuptake inhibitors and eased by these same drugs in a manner similar to the effects of these drugs on pain syndromes linked to peripheral neuropathy. There is growing evidence that some antimuscarinic drugs may promote peripheral nerve regeneration, as outlined below. This holds out the prospect that an antimuscarinic drug may yet be one of the better treatments for some of these urogenital states.

The overactive bladder treatment review cited above,^18^ cautioning against the use of drugs with anticholinergic effects, has all the hallmarks of marketing efforts to replace an older successful drug with a new drug, mirabegron in this case.^21^ The Australian regulator has stated that mirabegron, a beta-3 agonist, has little to recommend it.^22^ But more to the point, the suggestion in the review that mirabegron should be used because a drug with anticholinergic effects might lead to dementia after a relatively brief exposure makes little sense. Almost any drug can lead to confusion in older individuals, as can urinary tract infections. The evidence an anticholinergic drug is more likely to do this than an SSRI, an antibiotic, or a steroid, is weak.

As regards constipation, many antidepressants and antipsychotics with distinct catecholamine effects and minimal antimuscarinic effects such as mirtazapine cause constipation in a way that pure anticholinergic drugs do not.^23^ The American duloxetine product label states: the most commonly observed adverse reactions in Cymbalta-treated patients were nausea, dry mouth, somnolence, fatigue, constipation, decreased appetite and hyperhidrosis.^24^

The anticholinergic effects of many drugs have been blamed for falls, which again seems counter-intuitive in that these drugs are more likely to increase heart rates and raise blood pressure than to drop it. The catecholamine effects of many drugs that cause falls are more likely to cause these falls through sedative effects and an action on adrenergic receptors to cause orthostatic hypotension, than any anticholinergic properties are to cause falls.

Implicating anticholinergic drugs in TD^20^ also runs into problems in that the antipsychotics least likely to cause tardive dyskinesia, clozapine, thioridazine, quetiapine and olanzapine have the clearest antimuscarinic effects. There is no evidence primary anticholinergic agents, such as benztropine or procyclidine, cause TD, other than in the past perhaps by making megadoses of antipsychotics more tolerable.

As regards, any cognitive effects of current drugs with minimal or no anticholinergic actions being attributed, nevertheless, to their anticholinergic effects, one of the better examples of this comes from a Food and Drug Administration (FDA) review of an application to license brexpiprazole for agitation linked to Alzheimer’s dementia,^25^ in which the review states:

Safety findings from studies evaluating . . . citalopram, escitalopram, sertraline for BPSD symptoms have reported adverse events (AEs) consistent with their use in elderly patients including worsening cognitive function and anticholinergic effects and an increased incidence of gastrointestinal symptoms and QT prolongation.^26,27^

Consulting the references cited, the only side effects listed are cognitive, gastric, QT prolongation and falls. The cognitive effects in these trials did not stem from an anticholinergic action. It is unlikely that the falls did. And citalopram and escitalopram in particular come with warnings for QT prolongation where antimuscarinic agents do not.

In addition, although largely marketing copy, when the atypical antipsychotics were being promoted, these treatments that have marked antimuscarinic properties were sold as better for patients’ cognitive state than other antipsychotics.^28^ Beyond the marketing copy, as antimuscarinic agents are effective antiparkinsonian treatments, they can be expected to improve cognitive function in many patients with this condition or drug-induced states, if only by reversing cognitive slowing.

The idea that the cholinergic system underpins dementia has also not worked out.^29^ While a generation of cholinomimetic drugs from tacrine to donepezil have demonstrable benefits on activities of daily living scales in dementia, these are minor effects, differing little from the effects of nicotine, and not linked to any change in the course of any dementia.

Marked anticholinergic overload, as in henbane consumption, is linked to confusion, but delirium differs from dementia and relatively minor doses of steroids can equally cause delirium.^30^

It is also easier to demonstrate substantial episodic memory and other cognitive problems on benzodiazepines than on anticholinergic drugs, and these drugs are also recognized as causing delirium in the elderly in clinical doses.^31^

Significant delirium also arises with dopaminergic drugs, in the form of neuroleptic malignant syndrome,^32^ and with the serotonin syndrome linked to SSRIs and related drugs.^33^

As regards vision, antimuscarinics do dilate pupils and impair accommodation. These effects are immediate, useful and reversible. In contrast, SSRIs, devoid of anticholinergic effects, cause marked visual effects such as visual snow and night blindness, which often get worse on withdrawal and do not remit when treatment stops.^34^

On other domains such as sexual function, the significant problems that antidepressants trigger stem primarily from their serotonergic and catecholaminergic effects rather than any cholinergic effect.^35^ Drugs active on the cholinergic system have for the most part beneficial effects on sexual function.

In terms of these problems at least, the conventional story about the anticholinergic drugs appears overplayed. There are adverse effects, but little evidence that these effects are more serious than effects mediated through the serotonin, catecholamine or other systems. But in addition, we have lost sight of some beneficial effects of an anticholinergic action on mood, for instance.^36,37^

## Older anticholinergic benefits

Trials of atropine in melancholia (severe depression) and other anticholinergic drugs in depression^38^ have pointed to a benefit where SSRIs are close to completely ineffective in melancholia.^39,40^ This is consistent with the experience of patients. When anticholinergic drugs were more regularly used, patients would often prefer to continue with these drugs, which they found more tranquilizing than the antipsychotics or other medication they may be on. This is true also of patients using these drugs for Parkinson’s disease and hyperhidrosis.^41^

Similarly, clinical trials have repeatedly demonstrated that the TCAs are more effective antidepressants than the SSRIs.^42,43^ Rather than being pure drugs, the TCAs combine therapeutic principles, one of which is an anticholinergic action, which in line with the point about euphoria above has at least as clear a potential to help patients as any vigilance-enhancing action mediated through the catecholamine system or serenic effect mediated through the serotonin system.^37^

Finally, while there can be a rebound syndrome linked to stopping anticholinergic drugs, this is thought to last ordinarily a matter of 48 h or so, similar to the rebound linked to stopping beta-blockers.^44^ This rebound syndrome is less problematic clinically than the dependence linked to antidepressants, which is a greater hurdle to efforts to reduce medication burdens.

These older studies reveal that before the catecholamine hypothesis, the idea that an antimuscarinic agent might have an antidepressant effect did not appear unreasonable. More recent studies indicate that the antidepressant bupropion has an antinicotinic effect.^45^ In addition, an older drug, dextromethorphan, has recently attracted interest as a possible antidepressant,^46^ and it has both antinicotinic and antimuscarinic effects.^47^

## Newer anticholinergic benefits

Recent research points to possible benefits of antimuscarinic agents in the treatment of multiple sclerosis (MS). In 2012, a Scripps Institute study indicated benztropine could stimulate remyelination of nerve fibres.^48,49^ The view at that time appeared to be that there might be some other underlying effect, which if discovered might permit the company to bring this new therapeutic principle on the market at a profitable price, but its antimuscarinic effect now looks key.

Since then, the antimuscarinic agent, clemastine, has been identified as a remyelinating drug in MS.^50,51^ Clemastine is currently being tested in several phase II clinical trials in persons with relapsing-remitting MS.^52^ Convincing evidence has also emerged that pirenzepine, a quaternary amine anticholinergic agent, that does not cross the blood–brain barrier can stimulate regrowth of peripheral small nerve fibres in animals.^53–55^

Phase II clinical studies using a topical formulation of pirenzepine are under way in mild-to-moderate neuropathy and type 2 diabetes.^56^ Studies have also begun in patients suffering from chemotherapy-induced peripheral neuropathy.^57^

It is not yet clear how these benefits arise. It might be through an enhancement of angiogenesis and blood flow to deprived areas with nerve fibres following blood vessels. Another angle is that antimuscarinic agents can reverse mitochondrial problems in nerve fibres, which caused them to atrophy in the first instance.

To date, most other psychotropic drugs appear to cause nerve fibre damage. The antipsychotic group of drugs is linked to a loss of brain cells on brain scans^58^ and linked to tardive dyskinesia and other tardive neurological syndromes arising during ongoing treatment or attempted withdrawal from treatment^59^ and more generally linked to a loss of two decades of life expectancy when used chronically.^60^

The antidepressant, anticonvulsant and benzodiazepine group of drugs are used in the management of pain syndromes linked to peripheral neuropathies, but some evidence suggests the benefit they confer may stem from further damage to peripheral nerve endings^61^ rather than a conventional analgesic effect.

The regenerative effects of antimuscarinic agents on peripheral nerves, especially on small fibres, may also be of benefit for the protracted withdrawal syndromes linked to antidepressants and antipsychotics that happen for some people, possibly with a pre-existent vulnerability. Post-SSRI sexual dysfunction (PSSD) offers one example of this,^35^ and there are also enduring visual effects after withdrawal.^34^

## Discussion

The idea of a Magic Bullet that corrects a known chemical abnormality, without collateral damage, is the dominant therapeutic metaphor of our era. This is the case perhaps most obviously, and surprisingly, for the effects of drugs on behaviour. At present, antimuscarinic agents or the antimuscarinic effects of other drugs come close to being a polar opposite, a Maleficent Bullet, something that can only harm.

There are, however, few Magic Bullets, especially when it comes to behaviour. Most remedies offer a therapeutic principle that has been optimized, such as the anxiolytic effect of SSRIs, vigilance-enhancing effect of noradrenaline reuptake inhibitors, or tranquilizing effect of antipsychotics. This therapeutic principle can be helpful across a number of different syndromes. It will also suit some but not all patients. SSRIs are, for instance, the first-line treatment for obsessive-compulsive disorder (OCD). But their anxiolytic effect does not work for or suit all patients. Nicotine works for some patients intolerant of this effect.^62,63^ The idea of a therapeutic principle led Arvid Carlsson to create the first SSRI, for its anxiolytic effect. It also led him to note the benefits of nicotine for some people with OCD.^62^

Patients, clinicians or others consulting Wikipedia to find out more about diphenhydramine, a commonly used antihistamine, from which more selective monoamine reuptake inhibitors have come than from any other stem molecule, will find that it also has anticholinergic properties. These supposedly make it a deliriant.^64^ This is stated without any qualification in terms of dose.

If they check the University of North Carolina Eshelman School of Pharmacy guides for preventing falls in the elderly, they will find guidance to avoid antispasmodics because of their high degree of anticholinergic effects.^65^ The gut antispasmodics listed do not cross the blood–brain barrier. Other drugs listed as skeletal muscle relaxants, contain a mixed bag of older drugs that undoubtedly cause problems but not because they are anticholinergic.

A sedative-hypnotic group is listed as causing falls because of their highly anticholinergic properties, but six of the eight drugs in the group are barbiturates and the other two are antihistamines.

Even FDA echoes these claims.^25^ There may not be a single deprescribing guideline that does not stress that the single most important task is to reduce an anticholinergic burden.

Citing an anticholinergic effect apparently takes care of all harms with no requirement for those making the claim to point to any dose at which problems might appear, and no evidence that it is the anticholinergic rather than another effect of the drug that is causing the problem. Even the falls, dizziness and sedation lithium causes are linked to an anticholinergic effect without any evidence it acts on the cholinergic system.

One input to this state of affairs has likely come from marketing copy. Recently Ang, Horowitz and Moncrieff noted that although there was little evidence of lowered serotonin or a chemical imbalance in depression, the most cited articles in major journals on this topic mentioned the known lowering of serotonin in depression.^66^ In reviewing this article, I pointed out that a likely explanation was that the most cited articles were written by medical writers. Medical writers are adept at including tropes like a known chemical imbalance, and citing other articles that appear to be by other authors, but in fact the same writer or a colleague has written.

Regular citation by medical writers may have reinforced the idea of deleterious anticholinergic effects in a similar way. In the case of anticholinergic effects, these company articles are now supplemented by a growing literature on deprescribing, which although anything but ghostwritten cannot easily gainsay a literature that appears in some of the very best journals.

We can now recognize at least five muscarinic receptors.^67^ It seems unlikely that actions on all of these receptors will be problematic. Muscarinic system pharmacology is developing rapidly with an emphasis on both allosteric and orthosteric modulation, with efforts to find the appropriate balance between them.^68,69^ There is growing hope that these actions, that are very different to those of paroxetine and other potent and selective agents, will ground beneficial effects particularly on neural systems. If these drugs reverse neurodegenerative linked mitochondrial pathology, they may open a door to even more benefits.^70^

Extending the early research on the use of antimuscarinic drugs in peripheral neuropathy to SSRI withdrawal syndromes more generally, if shown to be of benefit, may revolutionize the way we see both anticholinergic drugs and other drugs in common use. If an anticholinergic drug, like pirenzepine, that does not cross the blood–brain barrier makes a difference to PSSD, which is viewed as a brain disorder, primarily because of its link to a drug labelled an ‘antidepressant’, this could transform the way we understand ourselves.

## Declarations

**Ethics approval and consent to participate:** Not applicable.

**Consent for publication:** Not applicable.

**Availability of data and materials:** Not applicable.

## References

[1] Loewi O. Über humorale übertragbarkeit der herznervenwirkung. Pflug Arch Ges Phys 1922; 193: 201–213.

[2] Healy D. The antidepressant era. Cambridge, MA: Harvard University Press, 1997.

[3] Raghavendra T. Neuromuscular blocking drugs: discovery and development. J Roy Soc Med 2002; 95: 363–367. 12091515 10.1258/jrsm.95.7.363 PMC1279945

[4] Nishtala PS Hilmer SN McLachlan AJ , et al. Impact of residential medication management reviews on drug burden index in aged-care homes: a retrospective analysis. Drugs Aging 2009; 26: 677–686. 19685933 10.2165/11316440-000000000-00000

[5] Koyama A Steinman M Ensrud K , et al. Long-term cognitive and functional effects of potentially inappropriate medications in older women. J Gerontol A Biol Sci Med Sci 2014; 69: 423–429. 24293516 10.1093/gerona/glt192 PMC3968824

[6] Salahudeen MS Duffull SB Nishtala PS. Anticholinergic burden quantified by anticholinergic risk scales and adverse outcomes in older people: a systematic review. BMC Geriatr 2015; 15: 31. 25879993 10.1186/s12877-015-0029-9 PMC4377853

[7] Kouladjian L Gnjidic D Chen TF , et al. Drug burden index in older adults: theoretical and practical issues. Clin Interv Aging 2014; 9: 1503–1515. 25246778 10.2147/CIA.S66660 PMC4166346

[8] Hilmer SN Mager DE Simonsick EM , et al. A drug burden index to define the functional burden of medications in older people. Arch Intern Med 2007; 167: 781–787. 17452540 10.1001/archinte.167.8.781

[9] Ailabouni NJ Mangin D Nishtala PS. Deprescribing anticholinergic and sedative medicines: protocol for a feasibility trial (DEFEAT-polypharmacy) in residential aged care facilities. BMJ Open 2017; 7: e013800 10.1136/bmjopen-2016-013800 PMC5775460 28416498

[10] Vogt M. The concentration of sympathin in different parts of the central nervous system under normal conditions and after the administration of drugs. J Physiol 1954; 123: 451–481. 13152692 10.1113/jphysiol.1954.sp005064 PMC1366219

[11] Pletscher A Shore PA Brodie BB. Serotonin release as a possible mechanism of reserpine action. Science 1955; 122: 374–374. 13246642 10.1126/science.122.3165.374

[12] Carlsson A . The rise of neuropsychopharmacology: impact on basic and clinical neuroscience. In: Healy D (ed.) The psychopharmacologists. London: Chapman & Hall, 1996, pp. 51–80.

[13] Schildkraut JJ. The catecholamine hypothesis of affective disorders: a review of supporting evidence. Am J Psych 1965; 122: 509–522. 10.1176/ajp.122.5.509 5319766

[14] Sandler M. Monoamine oxidase inhibitors in depression: history and mythology. J Psychopharm 1990; 4: 136–139. 10.1177/026988119000400307 22282941

[15] Moncrieff J Wesseley S Hardy R. Meta-analysis of trials comparing antidepressants with active placebos. Brit J Psychiatry 1998; 172: 227–231. 9614471 10.1192/bjp.172.3.227

[16] Kopelman M. The cholinergic neurotransmitter system in human memory and dementia: a review. Quart J Exp Psychol 1986; 38: 535–573. 10.1080/14640748608401614 3544081

[17] Beers M Ouslander J Rollingher I , et al. Explicit criteria for determining inappropriate medication use in nursing home residents. Arch Intern Med 1991; 151: 1825–1832. 1888249

[18] Zillioux J Welk B Suskind A , et al. SUFU white paper on overactive bladder anticholinergic medications and dementia risk. Neurourol Urodyn 2022; 41: 1928–1933. 36066046 10.1002/nau.25037

[19] Beder D Ashton P Mishra V. Overactive bladder in women. BMJ 2021; 375: e063526. 10.1136/bmj-2020-063526 34853012

[20] Chouinard G De Montigny C Annable L. Tardive dyskinesia and antiparkinsonian medication. Am J Psych 1979; 136: 228–229. 10.1176/ajp.136.2.228 32778

[21] Mintzes B Swandari S Fabbri A , et al. Does industry-sponsored education foster overdiagnosis and overtreatment of depression, osteoporosis and overactive bladder syndrome? BMJ Open 2018; 8: e019027. 10.1136/bmjopen-2017-019027 PMC5829862 29440213

[22] Therapeutic Goods Administration. Extract from the clinical evaluation report of mirabegron. Commonwealth of Australia, 2014, https://www.tga.gov.au/sites/default/files/auspar-mirabegron-140109-cer.docx

[23] Mirtazapine product label. Remeron (mirtazapine) tablets, https://www.accessdata.fda.gov/drugsatfda_docs/label/2010/020415s023s024.pdf (accessed 14 April 2023).

[24] Duloxetine product label. Cymbalta (duloxetine) tablets, https://www.accessdata.fda.gov/drugsatfda_docs/label/2010/022516lbl.pdf (accessed 14 April 2023).

[25] FDA briefing document NDA. 205422/S-009. Drug name: Brexpiprazole. Division of Psychiatry/office of Neuroscience, https://www.fda.gov/media/167066/download (accessed 14 April 2023).

[26] Porsteinsson AP Drye LT Pollack BG , et al. Effect of citalopram on agitation in Alzheimer disease: the CitAD randomized clinical trial. JAMA 2014; 311: 682–691. 24549548 10.1001/jama.2014.93 PMC4086818

[27] Seitz DP Adunuri N Gill SS , et al. Antidepressants for agitation and psychosis in dementia. Cochrane Database Syst Rev 2011: CD008191. 10.1002/14651858.CD008191.pub2 PMC12214452 21328305

[28] Juncos J Roberts V Evatt M , et al. Quetiapine improves psychotic symptoms and cognition in Parkinson’s disease. Mov Disord 2004; 19: 29–35. 14743357 10.1002/mds.10620

[29] Healy D. Notes towards a future history of treatments for cognitive failure. In: Ballenger J Whitehouse P Lyketsos C , et al. (eds) Treating dementia: do we have a pill for that?. Baltimore, MD: Johns Hopkins University Press, 2009, pp. 25–41.

[30] Schreiber MP Colantuoni E Bienvenu O , et al. Corticosteroids and transition to delirium in patients with acute lung injury. Crit Care Med 2014; 42: 1480–1486. 24589640 10.1097/CCM.0000000000000247 PMC4028387

[31] Gray SL Dublin S Onchee Yu O , et al. Benzodiazepine use and risk of incident dementia or cognitive decline: prospective population based study. BMJ 2016; 352: i90. 10.1136/bmj.i90 PMC4737849 26837813

[32] Berman B. Neuroleptic malignant syndrome. Neurohospitalist 2011; 1: 41–47. 23983836 10.1177/1941875210386491 PMC3726098

[33] Foong A-L Grindrod KA Patel T. Demystifying serotonin syndrome (or serotonin toxicity). Can Fam Physician 2018; 64: 720–727. 30315014 PMC6184959

[34] Healy D Lochhead J Mangin D. Development and persistence of patient reported visual problems associated with serotonin reuptake inhibiting antidepressants. Int J Risk Safety Med 2022; 33: 37–47. 10.3233/JRS-210018 34366298

[35] Healy D. Antidepressants and sexual dysfunction, a history. J Roy Soc Med 2020; 113: 133–135. 31972096 10.1177/0141076819899299 PMC7160790

[36] Healy D. Meta-analysis of trials comparing antidepressants and active placebos. Brit J Psychiatry 1998; 172: 232–234. 10.1192/bjp.172.3.227 9614471

[37] Healy D. Antidepressant psychopharmacotherapy at the crossroads. Int J Psych Clin Practice 1999; 3: S9–S15.

[38] Herz A. Central cholinolytic activity and antidepressant effect. Neuropsychopharmacology 1965; 4: 404–407.

[39] Hoch P Mauss W. Atropinbehandlung bei geisteskrankheiten. Arch Psychiat 1932; 97: 546–552.

[40] Kaspar S Moises H-W Beckmann H. The anticholinergic biperiden in depressive disorders. Pharmacopsychiatry 1981; 14: 195–198. 10.1055/s-2007-1019597 7323139

[41] Campanati A Gregoriou S Kontochristopoulos G , et al. Oxybutynin for the treatment of primary hyperhidrosis: current state of the art. Skin Appendage Disord 2015; 1: 6–13. 27172124 10.1159/000371581 PMC4857824

[42] Danish Universities Antidepressant Group. Citalopram: clinical effect profile in comparison with clomipramine: a controlled multicentre study. Psychopharmacology 1986; 90: 131–138. 2876451 10.1007/BF00172884

[43] Danish Universities Antidepressant Group. Paroxetine: a selective serotonin reuptake inhibitor showing better tolerance but weaker antidepressant effect than clomipramine in a controlled multicenter study. J Affect Disord 1990; 18: 289–299. 2140382 10.1016/0165-0327(90)90081-i

[44] Dilsaver SC Feinberg M Greden JF. Antidepressant withdrawal symptoms treated with anticholinergic agents. Am J Psych 1983; 140: 249–251. 10.1176/ajp.140.2.249 6849449

[45] Slemmer J Martin B Damaj M. Bupropion is a nicotinic antagonist. J Pharm Exp Ther 2000; 295: 321–327. 10991997

[46] Marwaha S Palmer E Suppes T , et al. Novel and emerging treatments for major depression. Lancet 2023; 401: 141–153. 36535295 10.1016/S0140-6736(22)02080-3

[47] Wright M Vann R Gamage T , et al. Comparative effects of dextromethorphan and dextrorphan on nicotine discrimination in rats. Pharmacol Biochem Behav 2006; 85: 507–513. 17112574 10.1016/j.pbb.2006.09.020 PMC1847596

[48] Deshmukh V Tardif V Lyssiotis C , et al. A regenerative approach to the treatment of multiple sclerosis. Nature 2013; 502: 327–332. 24107995 10.1038/nature12647 PMC4431622

[49] Cerles O Gonçalves T Chouzenoux S , et al. Preventive action of benztropine on platinum-induced peripheral neuropathies and tumor growth. Acta Neuropathol Commun 2019; 7: 9. 10.1186/s40478-019-0657-y PMC6337872 30657060

[50] Mei F Fancy SP Shen YA , et al. Micropillar arrays as a high-throughput screening platform for therapeutics in multiple sclerosis. Nat Med 2014; 20: 954–960. 24997607 10.1038/nm.3618 PMC4830134

[51] Mei F Mayoral SR Nobuta H , et al. Identification of the kappa-opioid receptor as a therapeutic target for oligodendrocyte remyelination. J Neurosci 2016; 36: 7925–7935. 27466337 10.1523/JNEUROSCI.1493-16.2016 PMC4961778

[52] Clemastine fumarate as remyelinating treatment in internuclear ophthalmoparesis and multiple sclerosis (RESTORE). ClinicalTrials.gov Identifier: NCT05338450.

[53] Carmine A Brogden R. Pirenzepine. Drugs 1985; 30: 85–126. 3928324 10.2165/00003495-198530020-00001

[54] Calcutt N Smith D Frizzi K , et al. Selective antagonism of muscarinic receptors is neuroprotective in peripheral neuropathy. J Clin Invest 2017; 127: 608–622. 28094765 10.1172/JCI88321 PMC5272197

[55] Jolivalt C Frizzi K Han M , et al. Topical delivery of muscarinic receptor antagonists prevents and reverses peripheral neuropathy in female diabetic mice. J Pharmacol Exp Ther 2020; 374: 44–51. 32327528 10.1124/jpet.120.265447 PMC7292964

[56] A 24-week study of topical pirenzepine or placebo in type 2 diabetic patients (T2DM) with peripheral neuropathy. ClinicalTrials.gov Identifier: NCT04005287.

[57] Han M Frizzi K Ellis R , et al. Prevention of HIV-1 TAT protein-induced peripheral neuropathy and mitochondrial disruption by the antimuscarinic pirenzepine. Front Neurol 2021; 12: 663373. 34211430 10.3389/fneur.2021.663373 PMC8239242

[58] Fusar-Poli P Smieskova R Kemptona MJ , et al. Progressive brain changes in schizophrenia related to antipsychotic treatment? Neurosci Biobehav Rev 2013; 37: 1680–1691. 23769814 10.1016/j.neubiorev.2013.06.001 PMC3964856

[59] Tranter R Healy D. Neuroleptic discontinuation syndromes. J Psychopharm 1998; 12: 306–311. 10.1177/026988119801200412 10065916

[60] Healy D Le Noury J Whitaker C , et al. Mortality for schizophrenia & related psychoses in two cohorts: 1875–1924 & 1994–2010. BMJ Open 2012; 2: e001810. 10.1136/bmjopen-2012-001810 PMC3488735 23048063

[61] Estebe J-P Myers R. Amitriptyline neurotoxicity: dose-related pathology after topical application to rat sciatic nerve. Anesthesiology 2004; 100: 1519–1525. 15166573 10.1097/00000542-200406000-00026

[62] Lundberg S Carlsson A Norfeldt P , et al. Nicotine treatment of obsessive-compulsive disorder. Prog Neuropsychopharmacol Biol Psychiatry 2004; 28: 1195–1199. 15610934 10.1016/j.pnpbp.2004.06.014

[63] Salín-Pascual RJ Basañez-Villa E. Changes in compulsion and anxiety symptoms with nicotine transdermal patches in non-smoking obsessive-compulsive disorder patients. Rev Invest Clin 2003; 55: 650–6544. 15011734

[64] Wikipedia. Diphenhydramine, https://en.wikipedia.org/wiki/Diphenhydramine (accessed 14 April 2023).

[65] University of North Carolina – Eshelman School of Pharmacy. High risk medication recommendations: a guide to evaluate fall risk in older adults, https://pharmacy.unc.edu/wp-content/uploads/sites/1043/2020/06/UNC-High-Risk-Medication-Recommendations_Final_8.5.19.pdf (accessed 14 April 2023).

[66] Ang B Horowitz M Moncrieff J. Is the chemical imbalance an ‘urban legend’? An exploration of the status of the serotonin theory of depression in the academic literature. Soc Sci Me Mental Health 2022; 2: 100098.

[67] Haga T. Molecular properties of muscarinic acetylcholine receptors. Proc Jpn Acad Ser B Phys Biol Sci 2013; 89: 226–257. 10.2183/pjab.89.226 PMC3749793 23759942

[68] Van der Westhuizen E Choy K Valant C , et al. Fine tuning muscarinic acetylcholine receptor signaling through allostery and bias. Front Pharmacol 2021; 11: 606656. 33584282 10.3389/fphar.2020.606656 PMC7878563

[69] Randáková A Jakubík J. Functionally selective and biased agonists of muscarinic receptors. Pharmacol Res 2021; 169: 105641. 33951507 10.1016/j.phrs.2021.105641

[70] Saleh A Sabbir M Aghanoori M , et al. Muscarinic toxin 7 signals via Ca2+/calmodulin-dependent protein kinase kinase β to augment mitochondrial function and prevent neurodegeneration. Molec Neurobiol 2020; 57: 2521–2538. 32198698 10.1007/s12035-020-01900-x PMC7253379

